# How lay people understand and make sense of personalized disease risk information

**DOI:** 10.1111/hex.12538

**Published:** 2017-01-17

**Authors:** Olga C. Damman, Nina M. M. Bogaerts, Maaike J. van den Haak, Danielle R. M. Timmermans

**Affiliations:** ^1^ Department of Public and Occupational Health Amsterdam Public Health research institute VU University Medical Center Amsterdam The Netherlands; ^2^ Department of Language, Literature and Communication VU University Amsterdam Amsterdam The Netherlands; ^3^ National Institute for Public Health and the Environment (RIVM) Bilthoven The Netherlands

**Keywords:** informed decision making, lay perspective, patient education, prevention, risk communication

## Abstract

**Background:**

Disease risk calculators are increasingly web‐based, but previous studies have shown that risk information often poses problems for lay users.

**Objective:**

To examine how lay people understand the result derived from an online cardiometabolic risk calculator.

**Design:**

A qualitative study was performed, using the risk calculator in the Dutch National Prevention Program for cardiometabolic diseases. The study consisted of three parts: (i) attention: completion of the risk calculator while an eye tracker registered eye movements; (ii) recall: completion of a recall task; and (iii) interpretation: participation in a semi‐structured interview.

**Setting and participants:**

We recruited people from the target population through an advertisement in a local newspaper; 16 people participated in the study, which took place in our university laboratory.

**Results:**

Eye‐tracking data showed that participants looked most extensively at numerical risk information. Percentages were recalled well, whereas natural frequencies and verbal labels were remembered less well. Five qualitative themes were derived from the interview data: (i) numerical information does not really sink in; (ii) the verbal categorical label made no real impact on people; (iii) people relied heavily on existing knowledge and beliefs; (iv) people zoomed in on risk factors, especially family history of diseases; and (v) people often compared their situation to that of their peers.

**Discussion and conclusion:**

Although people paid attention to and recalled the risk information to a certain extent, they seemed to have difficulty in properly using this information for interpreting their risk.

## 1 INTRODUCTION

Informing people about their personal risk plays a key role in the prevention of lifestyle‐related diseases, such as cardiovascular diseases (CVD), diabetes and chronic kidney disease (CKD).^1^, ^2^, ^3^, ^4^ For example, clinical guidelines in different countries stipulate that general practitioners practise cardiovascular risk assessment and communication.^5^, ^6^ Online personalized risk calculators are increasingly being used in this context, often as a first step in prevention programmes.^7^, ^8^ These risk calculators provide people with personalized information such as the risk factors (eg age, smoking, BMI) that modify their susceptibility, with numerical information about their likelihood of developing the illness within a particular time frame and with advice on how to reduce their risk. People are expected to use this information and obtain insight into their risk, thereby enabling them to make informed health‐related decisions, which would ultimately improve population health.^9^, ^10^

Precisely how an individual's personalized risk resulting from an online risk calculator should be communicated has become a crucial question.^8^, ^11^, ^12^ Although some risk formats (eg natural frequencies and some graphical formats in addition to numerical information) in general seem to evoke better risk understanding than other formats (eg percentages only),^13^, ^14^, ^15^ it remains unclear how the provided risk information supports an individual's understanding of their risk. Previous user tests have shown that risks presented as percentages in risk calculators often have unclear or ambiguous meaning for end‐users, even when accompanied by graphical information.^8^, ^16^, ^17^ Other more general problems revealed by such user tests are that the risk message does not necessarily match the individual's existing beliefs and expectations about risk factors, and that, perhaps partly as a result of this, many end‐users with relatively high risks tend to undervalue or normalize their risk.^17^, ^18^ Such problems are particularly urgent as many people, not only those with lower educational levels, have poor health literacy and numeracy skills,^19^, ^20^ thereby placing them at a higher risk of misinterpreting information and making non‐informed decisions.^15^ It is therefore important to investigate how end‐users of risk calculators make sense of their risk result and to improve risk communication accordingly.

To date, little qualitative work has been performed to investigate how people exactly understand risk information in risk calculators.^4^, ^8^, ^17^ Most of this research has employed think‐aloud protocols and/or user evaluations, but it can be questioned whether these methods fully capture how people understand risk information. User evaluations typically investigate the user‐friendliness of information from the perspective of end‐users themselves, which does not give a more “objective” assessment of how people understand information.^14^ Think‐aloud protocols provide insight into the thought processes of people who use information^21^, ^22^; although this can be useful in assessing how people understand the provided information, the method does not necessarily capture how people subsequently utilize this information to interpret and make sense of their risk. We therefore adopted a novel qualitative approach that followed different essential phases in the process of understanding risk information provided in a risk calculator. The aim of this study was to examine how lay people understand the result from the above‐mentioned online cardiometabolic risk calculator using eye tracking, a recall task and qualitative post‐test interview questions. We assumed that in order to understand their personal disease risk in a risk calculator, people have to (i) pay attention to essential information; (ii) be able to recall this essential information; and (iii) use this essential information in their risk interpretations. Previous qualitative studies did not adopt such a qualitative approach that specifically focused on these phases in the process of individual comprehension and interpretation, but rather focused on general reactions to provided information.

## 2 METHODS

### 2.1 Study design

**Figure 1:**
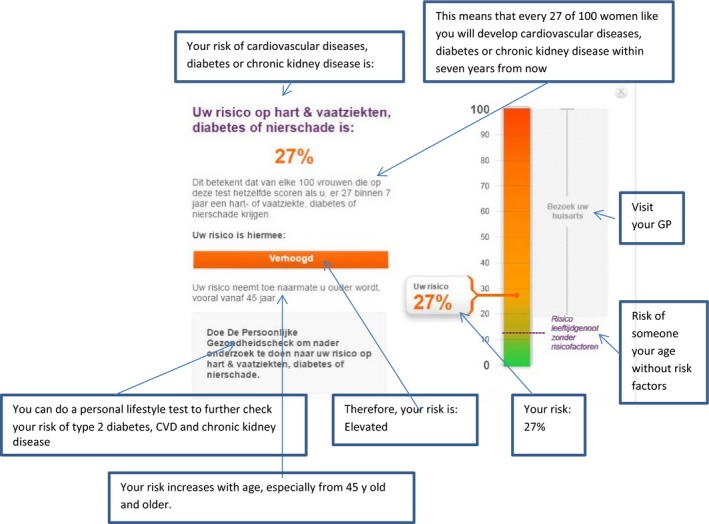
The risk communication in the risk calculator [Colour figure can be viewed at wileyonlinelibrary.com]

Our case study involved the online risk calculator that is part of the Dutch National Prevention Program for CVD, type 2 diabetes and CKD. This risk calculator, which is the first step in the prevention programme, can be used by general practitioners to identify high‐risk individuals. General practitioners can invite patients between 45 and 65 years of age to fill out the risk calculator at home for a first risk estimation based on sex, age, smoking status, BMI, waist circumference and family history of type 2 diabetes and CVD.^23^ Only individuals whose test results reveal an elevated risk are advised to see their general practitioner for further screening. Figure 1 shows the risk communication in the risk calculator. The study was exempted from review by a medical research ethics committee in accordance with local regulatory guidelines and standards for human subjects’ protection in the Netherlands (Medical Research Involving Human Subjects Act (WMO), 2005).

We performed a qualitative study consisting of three parts, corresponding to the assumed essential phases in the process of understanding risk information.

1. Attention: participants completed a risk calculator while an eye tracker (TOBII) registered their eye movements; the interviewer did not intervene in this phase.
2. Recall: participants were provided with a recall task after they had completed the risk calculator, assessing their recall of different parts of the risk information.
3. Interpretation: Semi‐structured questions were posed during a 30‐minute interview, focusing on participants’ subsequent risk interpretations.

We assumed that in order to understand their risk, people should, at a minimum: (i) pay attention to some of the numerical information to get an idea of the size of their risk^24^ and also of the verbal categorical label, bar graph or comparative risk information (the risk of someone of the same age without risk factors) that form part of the risk communication to provide intuitive or “gist” meaning of the number^25^; (ii) recall the size of their risk (ie in numbers) and some of the information aimed to provide intuitive meaning, for example the verbal label; and (iii) use this information as well as information about qualitative dimensions of risk (ie their personal risk factors, the controllability of their risk^26^, ^27^) to interpret and make sense of their risk result.

### 2.2 Recruitment and sample characteristics

We recruited people from the target population of the prevention programme (people aged between 45 and 60 years without a medical history of type 2 diabetes, CVD and CKD) through an advertisement placed in a free distributed local newspaper. This advertisement mentioned that the study would focus on people's opinions about health websites. A total of 21 people responded and were provided with further details. These 21 people were initially all willing to participate; 16 of them actually participated. The participants’ characteristics are presented in Table 1.

**Table 1:** Participants’ characteristics

Variable	N (%)
Age	
45‐50 years	6 (37%)
51‐55 years	6 (37%)
56‐60 years	1 (6%)
60‐65 years	3 (20%)
Gender	
Male	3 (19%)
Female	13 (81%)
Educational level	
Low (no or primary education)	1 (6%)
Medium (secondary education)	7 (44%)
High (tertiary education)	8 (50%)
Subjective numeracy^a^	M=3.5 (2.1‐5.1)
Subjective health literacy^b^	
Inadequate	10 (63%)
Adequate	6 (37%)
Result risk calculator	
No elevated risk	4 (25%)
Slightly elevated risk	10 (63%)
Elevated risk	2 (13%)

a Based on the eight subjective numeracy items developed by Fagerlin et al.^28^ All questions use 6‐point Likert‐type scales with endpoints as marked (1‐6). A higher score indicates a higher subjective rating of numeracy abilities and preferences.

b Based on the three subjective health literacy screening items developed by Chew et al.^29^: (i) “How often do you have someone help you read hospital materials?” (ii) “How confident are you filling out medical forms by yourself?” and (iii) “How often do you have problems learning about your medical condition because of difficulty understanding written information?”. Inadequate health literacy if answers other than “never” on items 1 or 3 and/or answers other than “extremely” or “quite a bit” on item 2.

### 2.3 Procedure

Participants were interviewed at the VU University. To make sure the participants would feel comfortable, the interviews were conducted in an attractive laboratory setting that was especially designed to facilitate laboratory research in a realistic environment. The interviewer (NB) informed participants about the online risk calculator and the aim of the interview and then instructed them about the use of the eye tracker and asked permission to audiotape the interviews. After providing written consent, the interviewer started the online risk calculator on the computer screen. Participants completed the risk calculator while an eye tracker registered their eye movements and fixations (part 1); in this phase, the interviewer sat a few metres behind the participant and did not intervene; she viewed the eye movements on a second screen. After completing the risk calculator, the interviewer provided the participant with a recall task (part 2, Section 2.4). Next, the interviewer conducted the semi‐structured interview using an interview guide (part 3, see Section 2.4). Finally, participants’ socio‐demographic characteristics (sex, age, educational level, language spoken at home), subjective numeracy^28^ and subjective health literacy^29^ were assessed in a short survey. Participants were thanked for their participation and were given a small financial reward (€20).

### 2.4 Materials

**Figure 2:**
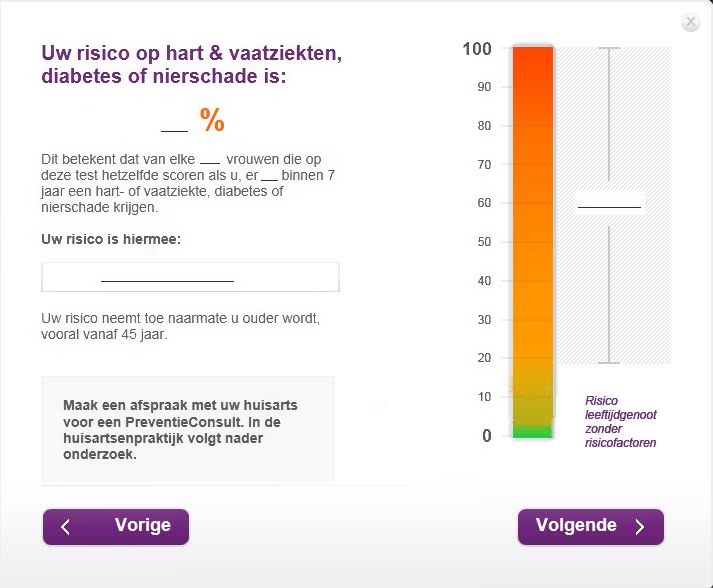
Blank hard copy page of the risk result as used in the recall task [Colour figure can be viewed at wileyonlinelibrary.com]

The online risk calculator communicates people's personalized risk in different formats on a single web page (Figure 1): a percentage (eg your risk is 14%), a natural frequency (eg 14 of 100 men/women like you will develop the diseases within 7 years from now), a bar graph, a categorical verbal label (eg your risk is “slightly elevated”) and comparative risk information (eg the risk of someone your age without risk factors is 10%). Information about the risk factors contributing to people's personal risk was provided on another web page. In part 2, we used a recall task that assessed participants’ recall of the personalized risk information as provided in Figure 1. A blank hard copy page of this web page was provided to participants (Figure 2) and they were asked to fill out their test results, that is the percentage, the natural frequency, the categorical verbal label and the statement on the right side of the bar graph.

Part 3 used an interview guide specifically designed to let participants elaborate on their risk interpretations and to make sense of their test result. We first asked questions about how people perceived their risk after receiving their test result. Examples included: “How do you interpret your risk of getting type 2 diabetes, CVD and CKD?” “How likely do you think you are to develop one of these diseases and why?” In the second part, we again provided people with their personalized risk on the computer screen and compared it to people's answers to the recall task. We asked participants explicitly about perceived difficulties in completing the recall task and then together reviewed the different parts of the risk communication. Examples of interview questions were as follows: “What were your thoughts on seeing your risk percentage?” “How do you feel about having a risk that is elevated/slightly elevated/not elevated?”

### 2.5 Data analysis

All interviews were audio‐recorded and transcribed verbatim. We analysed the eye‐tracking data by searching for patterns in the individual absolute gaze duration heat maps (showing the observed and unobserved areas^30^) and individual gaze plots (showing gaze motions as a sequence of saccades and fixations^31^) of the web page showing people's risk result. While these analyses are well suited to shedding some light on the attention paid by people to the information, one should remain careful in interpreting the data, because heat maps and gaze plots cannot fully stand on their own.^32^ In making inferences about the eye‐tracking patterns in relation to risk understanding, we compared the eye‐tracking data to the recall data and the qualitative themes.

Analysis of the interview data in MAXQDA followed the phases of thematic analysis as described by Braun and Clarke.^33^ Initially, three researchers (NB, OD and MH) read all transcriptions. Observations made by the interviewer (NB) during the interview about how respondents interpreted and used the risk information were discussed. Subsequently, all interviews were coded by two researchers independently (11 were performed by NB and OD, and five were performed by NB and MH). First, the interviews of two different participants were each coded openly by NB and OD (ie we assigned preliminary codes to text fragments). This open coding meant that we had no pre‐existing coding or classification scheme. After discussing these codes, the three researchers further coded the remaining interviews while being able to access the codes of the first two interviews in an Excel sheet. When differences between the codes of the different researchers occurred, these were discussed in consensus meetings in which the two researchers participated; sometimes, the code of one of the researchers was adopted, and sometimes, a new code was created. In all cases, we were able to reach consensus. Next, the data were axially coded (integrating codes in broader related concepts), which resulted in a hierarchical list of codes. Based on this, initial themes were defined by two researchers (NB and OD). These themes were discussed with the other members of the research team (MH and DT) and the eye‐tracking and recall data were used to further refine the themes where necessary.

## 3 RESULTS

We will first describe the attention paid by people to the information, followed by their recall of that information. Next, people's risk interpretations are described by means of five qualitative themes.

### 3.1 Attention for information

We used the eye‐tracking data of 11 participants; of the remaining five participants, the data were of insufficient quality due to failed calibration. Note that this only concerned the eye‐tracking data; the recall and interview data were complete and fully analysed for 16 participants. Supplementary file 1 provides one example of a heat map and one example of a gaze plot, both of which were quite representative for our participants. Supplementary file 2 provides three individual gaze plots of three different participants. Both the heat maps and the gaze plots revealed that, overall, participants most extensively looked at the numerical risk information (percentage and frequency) and also, to some extent, at the verbal label. Participants particularly looked at the natural frequency in detail: many participants read this information 2‐3 times, as the gaze plots showed, which may suggest that this was hard to understand. They paid less attention to the bar graph, which is an important result because the bar graph was explicitly meant as a graphical aid to provide intuitive meaning to the information. Participants did look at the comparative risk information, which was another attempt to provide such intuitive meaning (bottom of bar graph), although not very extensively. Notably, participants rarely exactly read out their own risk from the bar; although most participants did look at the percentage displayed next to the bar, they did not place it on a scale from 0% to 100% (see the examples in supplementary file 2).

### 3.2 Information recall

Table 2 presents the information recall of all participants. Percentages were overall remembered well, whereas natural frequencies and verbal labels were remembered less well. Only one person adequately recalled the statement at the bar graph (“visit your GP”).

**Table 2:** Recall of different parts of the risk information

Participant	Percentage	Natural frequency	Verbal categorical level	Statement at the bar graph (visit your GP)
1 (A001)	+	− Filled in nothing	− Filled in nothing	− Filled in nothing
2 (A003)	+	± Only filled in numerator and not the denominator	− Filled in “light risk” instead of “slightly elevated risk”	− Filled in nothing
3 (A004)	+	− Filled in nothing	− Filled in nothing	− Filled in nothing
4 (A005)	+	− Filled in 100 (numerator) and 100 (denominator)	+	− Filled in nothing
5 (A007)	+	+	+	− Filled in nothing
6 (A008)	− Filled in nothing	+	+	− Filled in her weight instead of the statement
7 (A009)	+	− Filled in 5 out of 7	− Filled in the percentage again	− Filled in nothing
8 (A010)	+	+	− Filled in “small risk” instead of “slightly elevated risk”	− Filled in nothing
9 (A011)	+	− Filled in nothing	− Filled in “marginal risk” instead of “not elevated”	− Filled in nothing
10 (A012)	− Filled in 60% instead of 23%	− Filled in nothing	− Filled in nothing	− Filled in nothing
11 (A013)	+	− Filled in nothing	− Filled in “small risk” instead of “slightly elevated risk”	− Filled in nothing
12 (A015)	+	+	+	− Filled in nothing
13 (A017)	+	− Filled in nothing	− Filled in nothing	− Filled in nothing
14 (A018)	+	+	− Filled in nothing	− Filled in nothing
15 (A019)	+	− Filled in nothing	− Filled in “light risk” instead of “slightly elevated risk”	− Filled in nothing
16 (A020)	+	+	+	+

+, correctly recalled; −, incorrectly recalled or not recalled.

### 3.3 Risk interpretation

We identified five qualitative themes related to how people interpreted and made sense of their personalized disease risk information. The next section describes these themes and their corresponding subthemes, illustrated by respondents’ quotes in Table 3.

**Table 3:** Qualitative themes relating to people's risk interpretations, illustrated by key quotes from participants

Qualitative theme	Participant's quote	Participant's characteristics
Theme 1: numerical information does not really sink in		
Subtheme 1a: struggling to comprehend and recall numerical information	<Response to the recall task>: “Yes I believe that was 23% or was that BMI, I think, oh I can't remember, I saw something like 23%, I think it was to do with BMI but not with heart and… (…) I'm not thinking straight here, ‘of every’… I don't get it. That has to be 23% here, or not, well, I don't remember… (…) Yes, 23%, that's almost one in four, or not? Well, I don't get it.”	Woman, 64 years of age, medium educational level, inadequate subjective health literacy, risk of 23% (elevated)
	<Response to the recall task>: “That was 5%, right? (short silence) Of every….hmm.. Of every so many so many women who have the same test result as you. 5 out of seven 7 or something? 7 years. It was something like 5 out of 7. I think, but I'm not sure. (laughs) It's 5%. Right? (..)) 5 out of 7 that makes no sense of course.”	Woman, 45 years of age, medium educational level, adequate subjective health literacy, risk of 5% (slightly elevated)
Subtheme 1b: risk undervaluation	I: “What does it mean to you, a 12% risk, given what you already knew? R: Nothing really, since I live healthily and eh I'm never ill so nothing really, I do browse lots of health websites and stuff like that but I'm never ill, so it really doesn't affect me what it says here, I'm never ill so I don't expect to be at risk.”	Woman, 58 years of age, low educational level, inadequate subjective health literacy, risk of 12% (slightly elevated)
	“It also says here that the older you get the more risk you run so if it begins with 45, I'm 52, well then it probably increases with 1% per year, so that means that if I'm 100, I'll still have 48 eh, and then I'm still on the side of the eh [the bottom of the bar graph]”	Woman, 52 years of age, high educational level, adequate subjective health literacy, risk of 7% (slightly elevated)
Theme 2: the verbal categorical label made no real impact on people	“Then it says here, this means your risk is? And oh, small, oh, that's what they mean, right? Yes, small.”	Woman, 53 years of age, high educational level, adequate subjective health literacy, risk of 13% (slightly elevated)
Theme 3: people relied heavily on knowledge and beliefs about risk factors		
Subtheme 3a: reliance on knowledge and beliefs about risk factors	“The only thing is, eh, lifestyle, eh, whether you exercise or not, whether you have a sedentary job or not, use drugs, smoke, and eh, your eating habits, those are the most important, I think it would be better to explore those in more detail than to ask about my length and eh waist circumference and things like that.”	Man, 49 years of age, medium educational level, inadequate subjective health literacy, risk of 6% (not elevated)
	“Other than that I do eat healthy food and stuff, so that makes you think that maybe this won't happen to me”	Woman, 58 years of age, low educational level, inadequate subjective health literacy, risk of 12% (slightly elevated)
Subtheme 3b: Reliance on knowledge and beliefs about (lack of) complaints	“Yes, this information isn't relevant to me, because I already knew that I'm not at risk, and eh, that there's nothing wrong with me, since you'd have to have complaints, and I never have complaints so really eh, yes for me this test has no relevance at all.”	Woman, 58 years of age, low educational level, inadequate subjective health literacy, risk of 12% (slightly elevated)
Subtheme 3c: Reliance on knowledge and beliefs about the diseases	“Yes, cos it's about these diseases and that happen to be diseases that I'm not at all afraid of but if it would be about cancer or something like that, yes, then if this would be the result or 20, then I'd go to the doctor tomorrow, it's just what eh, what frightens you. (…). This is less clear, if it mentioned that you, what those diseases, yes, what they can do to you and your body, then you might be more motivated, like being really motivated to do that test”	Woman, 54 years of age, medium educational level, inadequate subjective health literacy, risk of 7% (slightly elevated)
	“Heart I get, but cardiovascular diseases, that makes me think, what kind of diseases are they exactly and kidneys, I don't know, kidneys, yes, your kidneys are very important, but I know, dialysis, people who need dialysis, I know that salt is bad for your kidneys but for the rest I don't know much about it”	Woman, 45 years of age, medium educational level, adequate subjective health literacy, risk of 5% (slightly elevated).
	“Not everyone knows exactly what chronic kidney disease is, chronic kidney disease, what is chronic kidney disease, does that occur when you fall and damage your kidney.”	Woman, 61 years of age, medium educational level, inadequate subjective health literacy, risk of 23% (elevated)
Theme 4: people zoomed in on risk factors, especially family history of diseases		
Subtheme 4a: focus on a diverse range of risk factors, including factors that were not part of the risk calculator	“But I do think that if you occasionally, that you have a greater risk of getting something when you smoke.”	Woman, 64 years of age, medium educational level, inadequate subjective health literacy, risk of 23% (elevated)
	“And stress, and stress is unavoidable and that's a factor too.”	Woman, 45 years of age, medium educational level, adequate subjective health literacy, risk of 5% (slightly elevated)
	“[The risk] is not very high, but I think that since I'm not overweight, that that helps too. I think that if I were obese, that I'd have more chance of, that you'd have a higher risk.”	Woman, 53 years of age, high educational level, adequate subjective health literacy, risk of 13% (slightly elevated)
	I: “Yes, and do you have any idea why your risk is elevated? R: Yes, of course, because of stress.”	Woman, 61 years of age, medium educational level, inadequate subjective health literacy, risk of 23% (elevated)
Subtheme 4b: family history of diseases was a salient risk factor	“My risk is not that high. I don't smoke and neither of my parents have CVD; diabetes doesn't run in my family.”	Man, 49 years of age, medium educational level, adequate subjective health literacy, risk of 6% (not elevated)
	“I think my risk is larger since it runs in the family.”	Woman, 50 years of age, high educational level, inadequate subjective health literacy, risk of 6% (slightly elevated)
Subtheme 4c: interest in more information about risk factors	“Well, I think, I do take the result seriously, because it's not just made up, but I'd value it more if lots more factors were included.”	Woman, 52 years of age, high educational level, adequate subjective health literacy, risk of 7% (slightly elevated)
Theme 5: people often compared their situation to that of their peers	I: “And the percentage, was that new information for you? R: Yes, yes, I've never before…I'm also a bit curious… how do other people score? What does their graph bar look like?”	Man, 49 years of age, medium educational level, adequate subjective health literacy, risk of 6% (not elevated)
	“Yes, well, my husband will have a greater risk since he smokes a packet of cigarettes a day, you know, he'll have a greater risk of getting cardiovascular diseases and those kinds of things since he smokes so very much, I think his risk is greater than mine. (…) Yes, people who are overweight, whose lifestyle is inadequate, whose eating habits are unhealthy and who don't exercise enough, have a sedentary job, those people will have a greater risk of cardiovascular diseases, of course, and when you're overweight, then too.”	Woman, 58 years of age, low educational level, inadequate subjective health literacy, risk of 12% (slightly elevated)
	I: “Yes, ‘cos who would you think those 6 out of 100 people would be? R: Well, eh, yes, I think they're the people who are just very, eh very busy, people who are stressed which puts them at more risk.”	Woman, 55 years of age, medium educational level, inadequate subjective health literacy, risk of 6% (not elevated)

#### 3.3.1 Theme 1: numerical information does not really sink in

We found that many participants struggled to adequately comprehend and recall the numbers provided, including the probability information (subtheme 1a). Furthermore, even if people did focus to some extent on the numerical information, for example as a result of probing questions, many participants tended to undervalue these numbers and seemed to interpret the risk as less severe than medical experts (subtheme 1b), indicating that the numerical information did not fully sink in. Theme 1 was related to themes 3 and 4 in the sense that people did not rely heavily on the numerical information in making sense of their risk, but rather on other aspects such as information about risk factors (Theme 4) as well as on existing knowledge and beliefs about a number of topics (Theme 3). Eye‐tracking data and recall data showed that participants did look at and remember numerical information, which suggests that participants did process this information to some extent.

##### Subtheme 1a: struggling to comprehend and recall numerical information

Many people struggled with comprehending and recalling the provided numerical information correctly. This became clear from post‐test interview questions about how people interpreted the numerical information, but also from people's responses to the recall task. Some participants tried to unite the percentage and the frequency of the probability information, which often proved hard for them. In addition, probability information was also confused with other numerical information, such as the time frame of the risk (7 years) and people's BMI. It was noticeable, in this respect, that participants did adequately recall the risk percentage.

##### Subtheme 1b: risk undervaluation

It also became clear that participants perceived relatively high risks (eg ranging from 12% to 23%) as rather low, and saw no reason for worry. This seemed to occur partly because of difficulties in interpreting numerical information (subtheme 1a), but also because people used their own risk factors in risk interpretations more than the size of the risk (subtheme 3a).

People also spontaneously talked about reasons why they felt that their risk was actually different from what was communicated in the test, causing them to downplay the size of their risk. For example, some participants believed that because they felt healthy, the risk that was communicated to them was an overestimation of their actual risk. Another reason why risk undervaluation might have occurred can be ascribed to the way the risk was presented; for example, the bar graph ascended all the way up to 100%. Although a bar graph that goes up to 100% is of course an adequate way to present percentages, risks of 15% or 20%, which would be considered severe risks by experts, can seem minor because they are in the lower part of the bar.

#### 3.3.2 Theme 2: the verbal categorical label made no real impact on people

Like the numerical risk information, the verbal categorical risk label (either “elevated,” “slightly elevated” or “not elevated”) made no real impact on participants. From an expert/epidemiological perspective, these labels are important information because they form the basis for deciding who needs further screening and who does not. However, our participants did not seem to regard this label as an essential element of their test result and recall data showed that many did not recall the label. Several participants with a “slightly elevated risk” who did not recall their verbal label correctly thought their label was “minor” or “small”. This might be related to the fact that people tended to undervalue their risk (subtheme 1b).

#### 3.3.3 Theme 3: people relied heavily on existing knowledge and beliefs

We found that participants primarily relied on their existing knowledge and beliefs to interpret their risk, rather than on the actual risk information provided (see themes 1 and 2). Many participants used their knowledge about risk factors to make sense of their own risk (subtheme 3a). Many participants also used their perceived physical complaints (or lack thereof) to judge their susceptibility, rather than the size of the risk as communicated in the risk calculator (subtheme 3b). Related to subthemes 3a and 3b, a third subtheme was that people's perceived susceptibility also depended on beliefs about, or “images” of, the diseases (subtheme 3c).

##### Subtheme 3a: reliance on knowledge and beliefs about risk factors

All participants more or less knew which risk factors from the risk calculator would apply to them personally and would thus contribute to their risk. Many participants, both with relatively low and with relatively high risks, relied heavily on such own knowledge and beliefs about risk factors, rather than on the numerical risk information provided.

##### Subtheme 3b: reliance on knowledge and beliefs about complaints (or lack thereof)

Many participants also used their perceived complaints (or lack thereof) to judge their susceptibility. It seemed that many participants believed that as long as they felt healthy and were free from medical/physical complaints, their risk would be rather low. The risk information did not affect these beliefs and perceptions.

##### Subtheme 3c: reliance on knowledge and beliefs about the diseases

Participants’ perceived susceptibility also partly depended on existing beliefs about the diseases in the risk calculator, which were, in turn, related to how familiar these were to people. In general, participants thought that cardiometabolic diseases were not very severe, for example in comparison with cancer. Several participants also indicated, without being prompted, that they had no clear picture of cardiometabolic diseases, especially of type 2 diabetes and CKD.

#### 3.3.4 Theme 4: people zoomed in on risk factors, especially family history of diseases

Apart from using existing knowledge and beliefs about risk factors in interpreting their personal risk as provided in the risk calculator (subtheme 3a), participants in general made numerous spontaneous causal attributions to a diverse range of risk factors, included or not included in the risk calculator (subtheme 4a). Particularly, family history seemed to be salient in participants’ risk perceptions and interpretations (subtheme 4b). Another indication that people focused on risk factors was that they wanted to have more detailed information about risk factors (subtheme 4c).

##### Subtheme 4a: focus on a diverse range of risk factors, including factors that were not part of the risk calculator

Unsolicited, participants made causal attributions with respect to ageing, overweight/obesity, smoking and family history of type 2 diabetes and CVD (all part of the risk calculator), but also with respect to an unhealthy diet, lack of physical activity (not explicitly in the test but underlying factors of overweight/obesity) as well as to alcohol use and stress (not part of the risk calculator). Participants’ perceived susceptibility also largely depended on the presence or absence of such risk factors, including those not included in the test (eg blood pressure, stress).

##### Subtheme 4b: family history of diseases was a salient risk factor

In particular, family history appeared to be prominent in participants’ risk perceptions and interpretations. Both the presence and absence of a family history of cardiometabolic diseases were used in risk interpretation. Actual examples in the family led to a clearer picture of the diseases, compared with people who had no examples in the family. These examples were often not the direct relatives referred to in the test, but having such examples nevertheless contributed to perceptions of susceptibility.

##### Subtheme 4c: interest in more information about risk factors

People wanted to have more detailed information about risk factors, both those included in the risk calculator, especially lifestyle factors, and those not included.

#### 3.3.5 Theme 5: people often compared their situation to that of their peers

Participants spontaneously compared their risk to that of peers, to make sense of their own risk result. They had stereotypical beliefs about “which people have an elevated risk” and they compared their own risk to these stereotypes to judge the severity of their own risk. This tendency seemed to be related to a risk undervaluation (subtheme 1b), although not all participants who compared their risk to the risk of (hypothetical) other people undervalued their own risk. While participants were curious to know the risk of others, our eye‐tracking data showed that they hardly looked at the comparative risk information available in the bar graph (supplementary file 2).

## 4 DISCUSSION

This study aimed to examine how lay people understand and make sense of the result from a disease risk calculator. We combined eye tracking with a recall task and qualitative post‐test interview questions in a qualitative study using a Dutch cardiometabolic risk calculator. Our findings showed that when making sense of their risk, people did not make extensive use of the risk information provided. Neither the numerical risk information nor the categorical verbal labels seemed to make a real impact on people's risk perceptions and interpretations, although they did look at and recall this information to a certain extent. Instead, people primarily relied on existing knowledge and beliefs, for example about the presence or absence of risk factors or about the severity of diseases.

The finding that participants relied so heavily on existing knowledge and beliefs and that, as a result, their perceptions and interpretations seemed hardly affected by the information provided was significant. Although research about this topic is scarce (see review of Sheridan^2^), previous studies have shown that giving people information about their cardiovascular disease risk can alter their risk perceptions. One explanation for our finding that the risk information hardly affected people's perceptions might be that the risk communication from our case example was suboptimal, and did not effectively guide end‐users to essential information about the size and severity of their risk. We did find that people attended to and recalled aspects of the provided risk information, most notably the risk percentage, but this did not always occur without any difficulties. Although we do not know for sure why so few participants filled in the natural frequency in our recall task (eg it might be that they believed that they did not have to fill in this frequency because it is essentially the same information as the percentage), it was noticeable that this natural frequency was badly recalled and discussed by participants. Overall, indeed, people had difficulties with interpreting and providing meaning to the numerical information, a result which has previously been found in the general field of risk communication^34^ as well as in studies that specifically focused on online disease risk calculators.^4^, ^17^ It was noticeable that people made limited use of the verbal categorical label and the graphical bar chart, which are formats explicitly intended to provide intuitive meaning to the numbers. In addition, the comparative risk information was often neglected, while it obviously has the potential to provide meaning to people's personalized risk.^35^ Graphical risk formats do have the potential to attract end‐users’ attention^35^ and to support their understanding.^14^, ^36^ We can only speculate about the reasons why our participants discounted this information. It might be that people themselves did not experience any problems in comprehending the percentage, and therefore found it unnecessary to also view the bar graph that again emphasized the percentage. The neglect of the verbal categorical label could indicate that the label itself had insufficient meaning or that the display of the label was suboptimal (use of colour, font, shading, etc.). Another explanation may be that the other cues on the page distracted from the verbal message. More in general, the total amount of information on the page may have been too much for people, leading them to neglect some parts of it. It is interesting to note that most experiments testing the effects of visual formats typically use hypothetical scenarios, while in our study people were confronted with their own actual risk. This may make a difference regarding how people use particular pieces of information.

Participants’ reliance on existing knowledge and beliefs might also reflect a more inherent tendency in people that is not particularly related to how information is communicated. It is known that existing knowledge and beliefs in general are very influential in shaping people's perceptions of and reactions to health and illness.^37^ Particularly when new information does not fit existing knowledge and beliefs, as was the case for many of our participants, it is likely that new information does not have much value for people^38^ and that people become sceptical about it.^39^ Several previous studies testing different risk formats have also shown that both risk perceptions and subsequent decisions of people are more influenced by their perceptions of individual risk factors, the experience of symptoms, as well as by negative emotions than by the size of their risk.^4^, ^18^, ^40^, ^41^ Our findings indeed suggest that people have salient knowledge and beliefs about particular risk factors and health symptoms in general. New information about the size of cardiometabolic risk is probably interpreted in the light of already existing beliefs or “mental models”.

One particularly salient aspect of people's existing beliefs that might be interesting in the light of better risk communication was having a family history of diseases. Although the risk calculator provided reliable information by using single enquiries for family history, our participants were rather sceptical about this “small set” of questions. Furthermore, participants talked a lot about family history in their answers to interview questions about their risk interpretations, which indicated that family history largely influenced their perceived susceptibility. A similar finding has been previously reported in the context of a breast cancer risk calculator.^18^ A more detailed family history assessment might lead to better use of the risk information, because it directly increases the perceived relevance of information and, by doing so, probably also increases people's motivation to process the information.^42^ A previous study also revealed that a detailed familial risk questionnaire contributed to users’ risk acceptance and motivation to adapt healthier lifestyles among people with a positive family history.^43^ An important caveat is that putting more emphasis on family history of diseases in risk communication may have reverse effects for people without a family history of diseases who do have other risk factors. Previous studies have demonstrated that the absence of diseases in the family can lead to low perceptions of risk.^44^, ^45^

### 4.1 Strengths and limitations

Our study might be limited by the fact that participants were all people who were interested in health websites, as we recruited them through an advertisement that mentioned health websites. A further limitation was that the eye‐tracking data of several participants (N=5) were of insufficient quality due to failed calibration. We should thus be cautious in drawing firm conclusions about people's attention for risk information. Another limitation is that we did not ask participants about their knowledge or basic perceptions of risk before they completed the risk calculator. Had we done so, we might have gained more insight into how pre‐existing beliefs influenced people's risk interpretations. An important strength of this study is that we used a novel approach to test how people interpret and make sense of their disease risk, that is a combination of eye tracking with a recall task and qualitative post‐test interview questions. This approach provided us with valuable insights into how people used the risk information from the risk calculator and how they interpreted their risk of cardiometabolic diseases.

## 5 CONCLUSION

Although people pay attention to and recall risk information in an online risk calculator to a certain extent, they do not seem to optimally use it in their risk interpretations. Risk communication in an online disease risk calculator could be improved by building on people's existing knowledge and beliefs (eg about risk factors such as family history of diseases), by providing clear, more elaborate information about the diseases and using alternative graphical formats of numerical risk.
