# Supplementary material for: How lay people understand and make sense of personalized disease risk information
Source: Health Expect. 2017 Jan 17;20(5):973–83. doi: 10.1111/hex.12538 (PMC5600228; doi:10.1111/hex.12538)
Supplement: Supplementary file 1 [file HEX-20-973-s001.docx]

Supplementary file 1


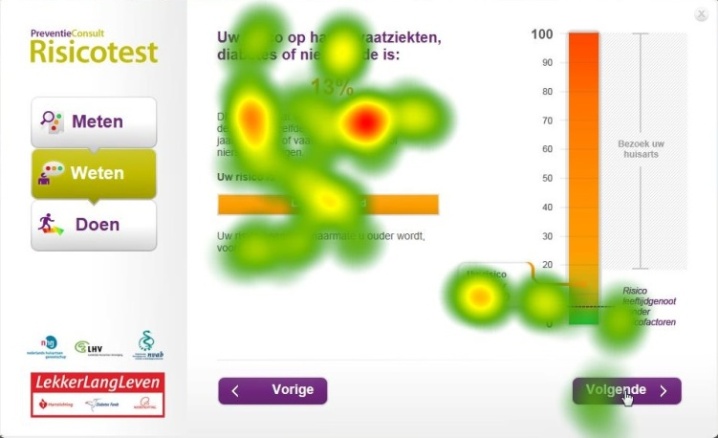


Figure : One example of a heat map of a participant.


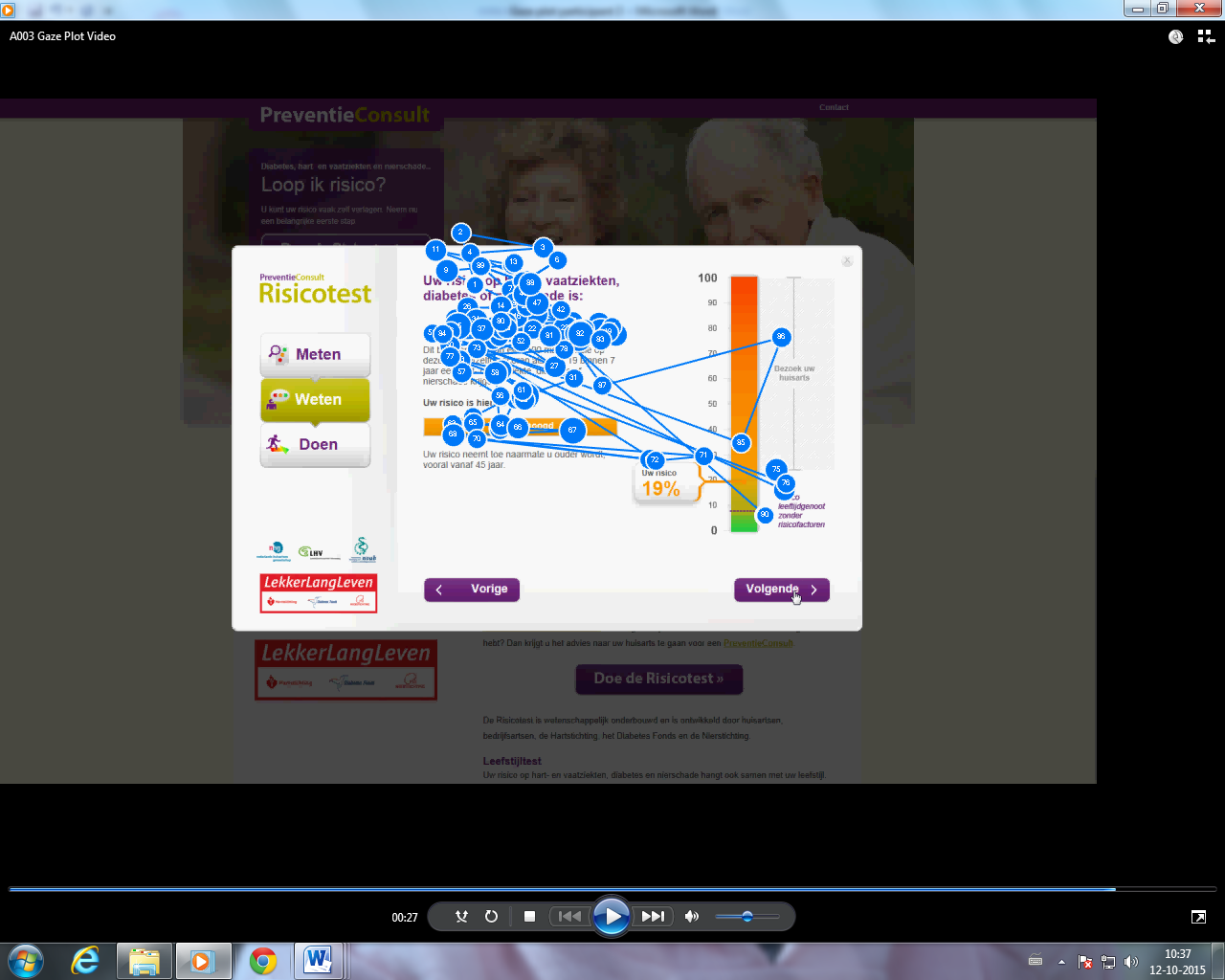


Figure: One example of a gaze plot of a participant.
