# Supplementary material for: How lay people understand and make sense of personalized disease risk information
Source: Health Expect. 2017 Jan 17;20(5):973–83. doi: 10.1111/hex.12538 (PMC5600228; doi:10.1111/hex.12538)
Supplement: Supplementary file 2 [file HEX-20-973-s002.docx]

Supplementary file 2


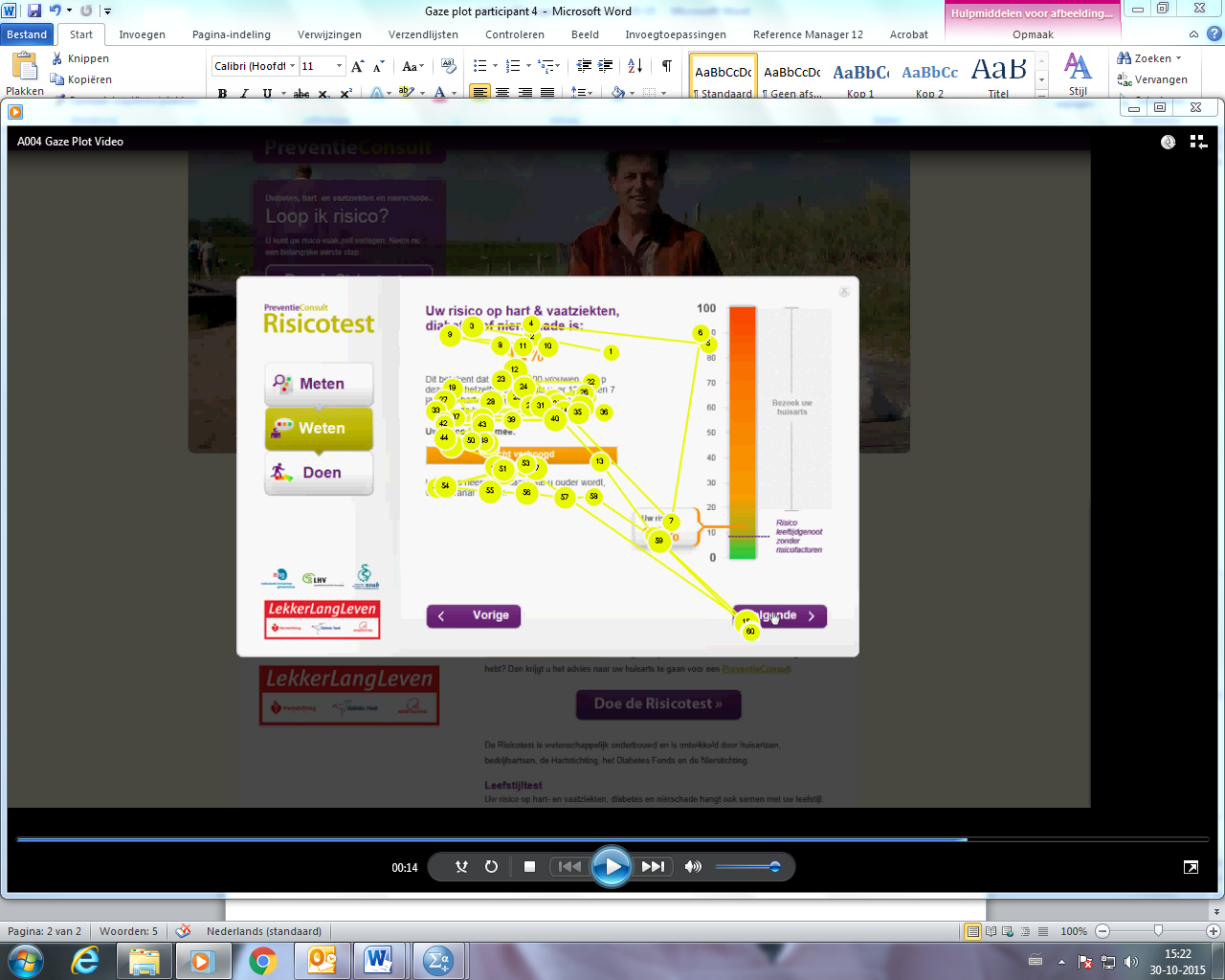


Figure: Gaze plot participant 4


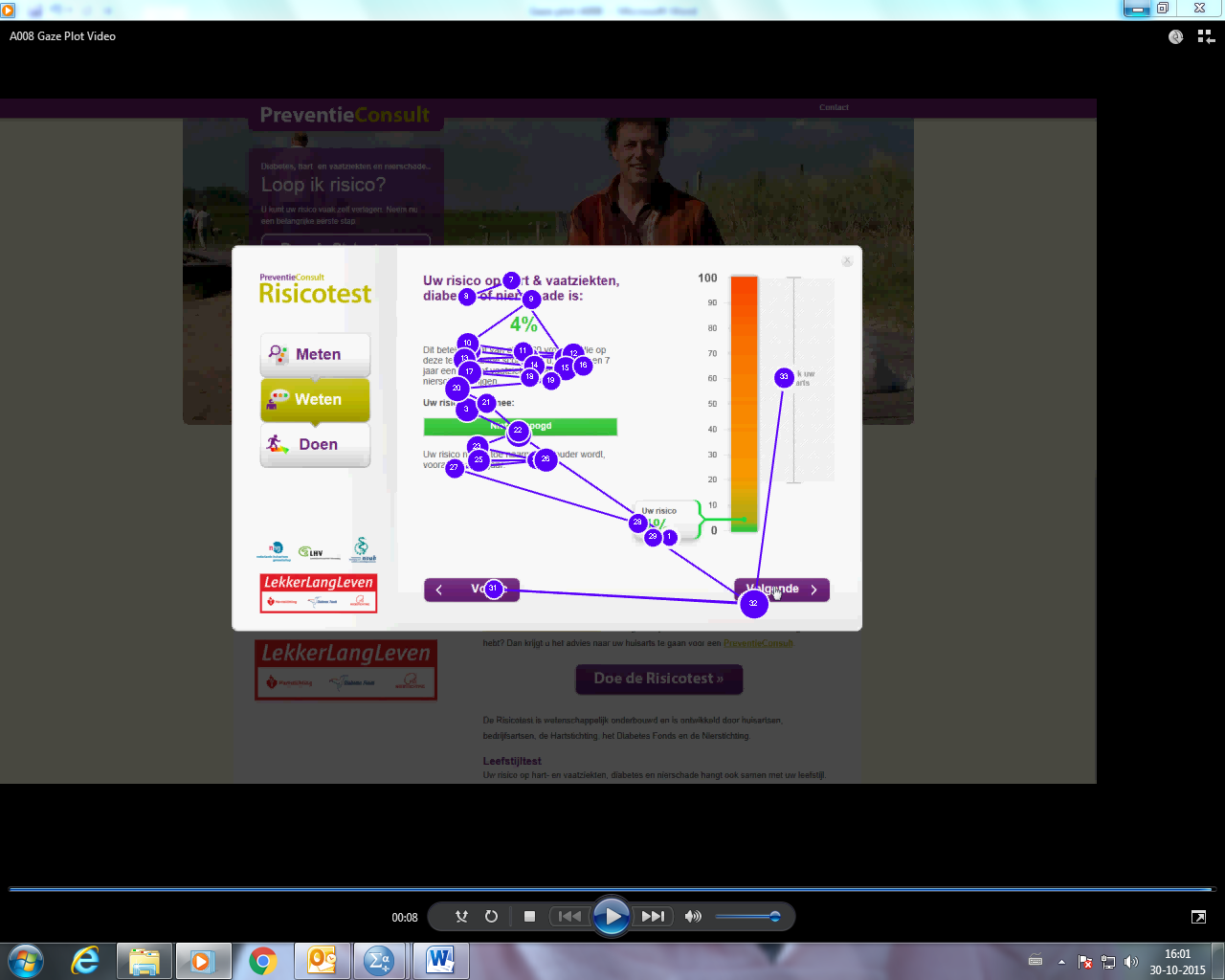


Figure: Gaze plot participant 8


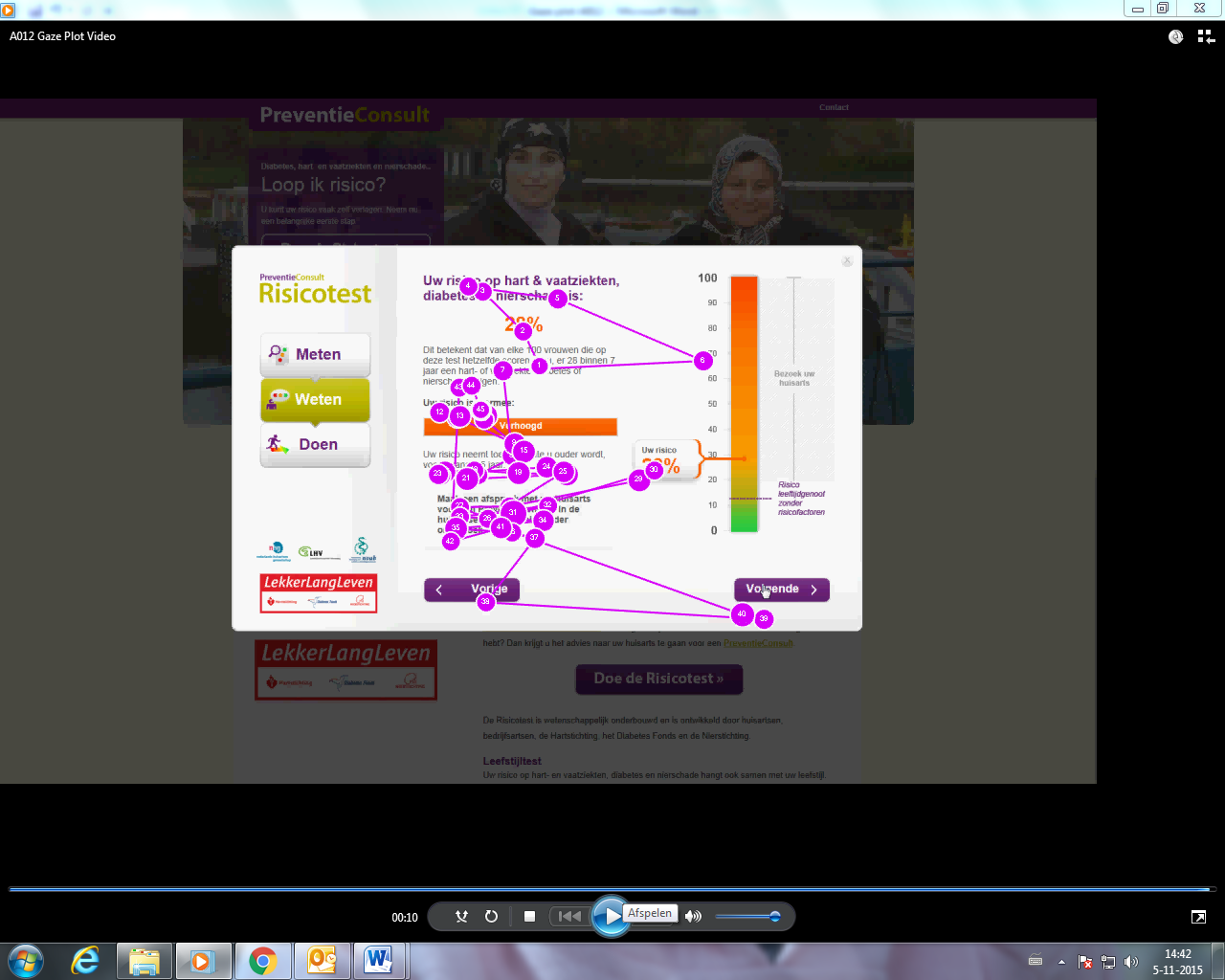


Figure: Gaze plot participant 12
